# Animal Models for Mental Disorders

**DOI:** 10.1007/s00702-023-02672-z

**Published:** 2023-08-14

**Authors:** Georg Juckel, Nadja Freund

**Affiliations:** Department of Psychiatry, Psychotherapy and Preventive Medicine, LWL University Hospital, Ruhr University, Bochum, Germany

In this special issue, several aspects of animal models advancing our knowledge of mental disorders are covered. In history, there were relevant findings from animal research for psychiatry. The 'sedative' effect of lithium was first observed in guinea pigs. Initial tests with benzodiazepines showed sedative, muscle relaxant and anticonvulsant effects in mice, and a taming effect was reported in monkeys and lions. The fact that prolonged use of benzodiazepines can cause the development of tolerance was also first detected in animal studies. Even electroconvulsive therapy (ECT) was established in pigs and dogs. Mechanisms of extinction as an important application in psychotherapy have been largely studied in animal experiments. The main problem with animal studies is the transfer to the human situation especially in disease. Therefore, validity criteria of animal models were developed: (A) Face validity: Equality or comparability of humans and animals: same stimuli lead to the same responses. (B) Predictive validity: Prediction that a clinically effective medication will lead to changes in the behavior of the animal model analogous to its effect in humans. (C) Construct Validity: The neuropathological constructs that are thought to be responsible for the development of disease symptoms can also be represented in the model, e.g., by brain lesions. The prepulse inhibition (PPI) to an acoustical startle response e.g. demonstrates these validity criteria. Face Validity is given as PPI can be reliably triggered in many mammals and even birds. Various experimental interventions (pharmacological e.g. by NMDA antagonists, lesions in cortico-limbic-striatal network, rearing in social isolation) lead to a reduction of PPI similar to that in schizophrenic patients and therewith construct validity is given. Last but not least, predictive validity is confirmed as typical and atypical neuroleptics lead to the normalization of PPI.

Modern in vitro and in silico methods allow the characterization of drugs regarding receptors, and toxicity even without the use of animal models questioning whether or not animal experiments for the development of psychopharmacological agents are still up to date.

However, several aspects still make the use of animal models necessary. They exclusively allow the closer investigation of the following aspects contributing to pathological behaviors: (a) brain loops: e.g. microdialysis. (b) behavioral tests: healthy and e.g. genetically manipulated animals; selective “symptoms”. (c) systemic failures: developmental animal models.

As long as psychopharmaceuticals cannot be developed on the “drawing board” (computers, cells) alone, animal models are needed and should act as precisely as possible in the organism and in diseases, should be used to investigate systemic side effects, to learn about the influence of not so well-known transmitters, loops, behavioral aspects, etc. Working with animal models has to be conducted with the problems of transferability in mind and a focus on the three R (reduction, refinement, replacement). However, at the current stage of research they continue to be necessary in psychopharmaceutical-related and other basic research.

 Rodent models displaying the specific phenotype resembling mental disorders are mainly generated by pharmacological, genetic or environmental manipulation.

An example of genetic manipulation is given in the article **Knock-out of the critical nitric oxide synthase regulator DDAH in mice impacts amphetamine sensitivity and dopamine metabolism** written by Kozlova and colleagues. Imbalances in the nitric oxide (NO) pathway have long been associated with psychiatric disorders. In the presented study mice lacking the enzyme dimethylarginine dimethylaminohydrolase 1 (DDAH1), which plays a central role in the NO pathway, are investigated. Increased novelty-seeking, reduced amphetamine-induced locomotion and abnormalities in the dopamine system indicate that the knockout of DDAH displays aspects of several psychiatric disorders rather than inducing one specific phenotype.

The article **Molecular signature of excessive female aggression: study of stressed mice with genetic inactivation of neuronal serotonin synthesis** by Strekalova and colleagues combines genetic manipulation, namely a partial inactivation of the tryptophan hydroxylase-2, with exposure to a stressful environment. Mutant female mice that were faced with a predator displayed higher aggressive behavior following the stress induction. In addition, deep sequencing of the prefrontal cortex revealed altered expression in the stressed mutant compared to wildtype mice in pathways associated with neurodevelopment, cognition and emotion regulation.

Another article addressing stress exposure has the title **Long-term effects of chronic stress models in adult mice.** Here, the authors Tran and Gellner give a comprehensive overview of the consequences of different chronic stressors including social stressors, restrained stress and a combination of aversive physical stimuli in the so-called chronic unpredictable mild stress paradigm. This overview is novel and relevant as it focuses on long-term consequences while often attention is only paid to short-term effects. From a transitional point of view, however, the long-lasting effects are most relevant and should be studied more intensively.

One consequence of stress exposure is also altered hemispheric asymmetries as discussed in the article **Hemispheric asymmetries in mental disorders: Evidence from animal studies** by Mundorf and Ocklenburg. After careful evaluation of multiple articles on the topic authors conclude that increased right hemispheric activity and left-biased behavior is associated with stress and accordingly negative mood. In addition, models for addiction, schizophrenia and neurodegenerative disorders have been associated with atypical asymmetries. Taken together, the animal models allow an in-depth evaluation of hemispheric asymmetries to draw conclusions for the human condition to potentially detect early alterations.

Long-term consequences of early life stress are in addition covered in the article **Maternal separation and its developmental consequences on anxiety and parvalbumin expression in the amygdala** by Abraham and colleagues. Here, separation from the mother in the early postnatal phase serves as a stressor and consequences are investigated during adolescence as well as early adulthood. Increased anxiety could be found in both age groups and imbalances in the GABAergic system might be one mediator. Interestingly, the trend for a reduced number of GABAergic cell could only be found during adolescence and might, therefore, serve as an early predictor.

Separation from the mother as well as an infection of the mother during pregnancy effects the development of white matter. The article **Oligodendrocytes Matter – A review of animal studies on early adversity** by Abraham and colleagues summarizes the impact of these two early aversive events on oligodendrocyte differentiation and maturation. Resulting increased cell death, simpler morphology and inhibited maturation are region specific to main regions with ongoing development affected. While exposure to adversity early in life has the highest impact, oligodendrocytes can even be affected by stressors later in life and long-lasting effects might predispose for psychiatric disorders.

Maternal immune activation is also covered in the article **Microglia and microbiome in schizophrenia – can immunomodulation improve symptoms?** by Juckel and Freund. Here, aspects of neuroinflammation in schizophrenia with a focus on gut-brain interactions are discussed. Evidence of disrupted microglia and microbiota in patients as well as animal models is presented. From a perspective, anti-inflammatory treatment options are presented and evaluated.

Alternative treatment options influencing the microbiom are also discussed in the article **Ketogenic diet for mood disorders – from animal models to clinical application** by Smolensky and colleagues. There is no doubt about the effect of diet on mental health, however, underlying mechanisms are often not clear. The article puts evidence from animal models together on how the high-fat low carbohydrate diet acts neuroprotective, anti-inflammatory and influences certain brain circuits to reduce depressive-like behavior.

Against this background, we believe that these articles from members of the German Society of Biological Psychiatry were able to show the possibilities and impact of animal models in psychiatry and clinical neuroscience.

